# The lysine methyltransferase Ehmt2/G9a is dispensable for skeletal muscle development and regeneration

**DOI:** 10.1186/s13395-016-0093-7

**Published:** 2016-05-27

**Authors:** Regan-Heng Zhang, Robert N. Judson, David Y. Liu, Jürgen Kast, Fabio M. V. Rossi

**Affiliations:** The Biomedical Research Centre, The University of British Columbia, Vancouver, Canada

**Keywords:** Euchromatic methyltransferase, Ehmt2, Ehmt1, G9a, GLP, Myogenesis, Skeletal muscle, Development, Regeneration, Myod

## Abstract

**Background:**

Euchromatic histone-lysine *N*-methyltransferase 2 (*G9a*/*Ehmt2*) is the main enzyme responsible for the apposition of H3K9 di-methylation on histones. Due to its dual role as an epigenetic regulator and in the regulation of non-histone proteins through direct methylation, G9a has been implicated in a number of biological processes relevant to cell fate control. Recent reports employing in vitro cell lines indicate that *Ehmt2* methylates MyoD to repress its transcriptional activity and therefore its ability to induce differentiation of activated myogenic cells.

**Methods:**

To further investigate the importance of G9a in modulating myogenic regeneration in vivo, we crossed *Ehmt2*^*floxed*^ mice to animals expressing Cre recombinase from the *Myod* locus, resulting in efficient knockout in the entire skeletal muscle lineage (*Ehmt2*^*ΔmyoD*^).

**Results:**

Surprisingly, despite a dramatic drop in the global levels of H3K9me2, knockout animals did not show any developmental phenotype in muscle size and appearance. Consistent with this finding, purified *Ehmt2*^*ΔmyoD*^ satellite cells had rates of activation and proliferation similar to wild-type controls. When induced to differentiate in vitro, *Ehmt2* knockout cells differentiated with kinetics similar to those of control cells and demonstrated normal capacity to form myotubes. After acute muscle injury, knockout mice regenerated as efficiently as wildtype. To exclude possible compensatory mechanisms elicited by the loss of G9a during development, we restricted the knockout within adult satellite cells by crossing *Ehmt2*^*floxed*^ mice to *Pax7*^*CreERT2*^ and also found normal muscle regeneration capacity.

**Conclusions:**

Thus, *Ehmt2* and H3K9me2 do not play significant roles in skeletal muscle development and regeneration in vivo.

**Electronic supplementary material:**

The online version of this article (doi:10.1186/s13395-016-0093-7) contains supplementary material, which is available to authorized users.

## Background

The formation of skeletal muscle begins in the embryonic somites [1], which generate the primary myotome and the first primitive myogenic structure containing muscle progenitors. Morphogen gradients including sonic hedgehog (Shh) [2, 3] and Wingless (Wnt) [4] ensure initial myogenic specification by controlling the expression of myogenic regulatory factors (MRFs—Myf5, Myod, Myog, and Mrf4)—a conserved family of muscle-specific basic helix-loop-helix (bHLH) transcription factors responsible for myogenic lineage commitment and differentiation. Embryonic muscle progenitors migrate, expand, and undergo subsequent waves of myogenesis persisting through fetal and early neonatal development resulting in the formation of the different skeletal muscles of the adult.

A proportion of fetal myoblasts also become localized underneath the basal lamina of newly formed myofibers. These cells become specified as satellite cells—the quiescent, tissue resident stem cell of the skeletal muscle, identifiable by the expression of paired box transcription factor, paired box 7 (Pax7). Satellite cells are responsible for the regenerative potential of the muscle and, upon acute injury, break quiescence and mimic their developmental programs by expanding rapidly, upregulating MRFs and differentiating to form new myofibers. In mice, this process leads to near-complete tissue regeneration and restoration of muscle function.

Despite much knowledge about the key transcription factors regulating myogenesis, the epigenetic landscapes required for the control of gene expression in skeletal muscle differentiation remain less well understood. Epigenetic mechanisms including different modifications on the unstructured C-terminals (tails) of histone proteins have an increasingly appreciated role in controlling gene expression during myogenesis [5]. Some studies [6–8] have found that specific histone acetyltransferases can interact with *Myod* and acetylate histones associated with muscle-specific genes, thereby activating their transcription. Recent genome-wide analyses have uncovered dynamic epigenetic changes during myogenesis [9], including the loss of histone 2B (H2B) ubiquitination [10]. Chromatin immunoprecipitation (ChIP)-seq analyses have also revealed the importance of bivalent domains containing both H3K4me1 and H3K27ac in regulating muscle enhancers during myogenesis [9]. These data also point to Myod as playing a key role in the recruitment of chromatin-modifying enzymes and transcription factors to activate such enhancers [11, 12].

A less well-characterized histone mark in myogenesis is H3K9me2, produced by euchromatic histone-lysine *N*-methyltransferase 2 (*Ehmt2*) (MGI: 2148922), also known as *G9a*. This Su (var)3-9 and enhancer of zeste (SET) domain containing methyltransferase dimerizes with its close homologue *Ehmt1* (aka *Glp*) to induce H3K9me2 [13]. Knockout of *Ehmt2* leads to a global reduction of H3K9me2 levels and early embryonic lethality in mice, underscoring its importance and the fact that *Ehmt1* cannot fully compensate for its loss [14]. Conditional knockouts of *Ehmt2* also demonstrated its importance in germ cell development [15], heart development [16], lymphocyte development [17, 18], leukemia [19], drug addiction [20], cognition, and adaptive behaviors [21, 22]. H3K9me2 appears to occur on repressed genes in euchromatin, whereas H3K9me3 is also a repressive mark, but associated with pericentromeric heterochromatin [23]. The repressive function is led by recruitment of proteins such as HP1, which bind preferentially to H3K9me2 and establish a repressive chromatin conformation [15]. The SET domain in *Ehmt2* and other histone methyltransferases also have the potential to methylate non-histone polypeptides [24, 25], allowing possible regulation of the localization and activity of a variety on non-histone proteins [26]. *Ehmt2* contains an ankyrin domain, enabling protein–protein interactions [27] with a variety of partners including DNA methyltransferases (DNMT) [28], which provide an alternative mechanism for gene repression [29]. Finally, *Ehmt2* can act as a gene activator in addition to its repressive roles, by interacting with coactivators such as in nuclear receptor-mediated transcription regulation [23, 30].

Despite *Ehmt2* being capable of regulating a diverse range of cellular and biological functions, little is known about its role in skeletal muscle. Working with the widely used murine cell line C2C12, Ling et al. [31] recently highlighted *Ehmt2* as a possible regulator of myogenic differentiation. Using in vitro overexpression and knockdown strategies, *Ehmt2* was shown to act as an inhibitor of myotube formation. Biochemical analyses suggested that EHMT2 also has the capability to directly methylate MYOD at K104 [31], revealing a novel *Bhlhe41*/*Sharp1*-dependent mechanism inhibiting myogenesis that is controlled by *Ehmt2* through both direct modulation of MYOD and the repressive H3K9me2 modification of *Myod* target genes [32, 33]. In spite of these initial findings, whether *Ehmt2* plays an equivalent role in a more complex biological system such as in vivo skeletal muscle development or regeneration is yet to be evaluated. In this study, we generated transgenic mouse strains to genetically delete *Ehmt2* during muscle development as well as in adult satellite cells. We found that proliferation and differentiation of satellite cells was not influenced by the absence of *Ehmt2*. Knocking out *Ehmt2* also failed to result in significant consequences for skeletal muscle development and in adult muscle regeneration in vivo*.* Thus, *Ehmt2* is completely dispensable for the normal functioning, maintenance, and damage response of murine skeletal muscle.

## Methods

### Mice and animal care

C57BL/6 mice harboring the *Ehmt2*^*floxed*^ allele were previously generated by Lehnertz et al. [17]. In this strain, Cre-mediated targeting of the *Ehmt2*^*floxed*^ allele results in a genomic deletion from exon 4 to exon 20 and a frameshift mutation that places the downstream coding sequence out of frame. All other transgenic strains used herein were generated by other groups [34–36] and obtained from The Jackson Laboratory. Inducible Cre recombinase was activated by intraperitoneal injection of tamoxifen dissolved in corn oil (250 mg/kg of body weight per day) for 5 consecutive days, followed by 7 days without any treatment to allow sufficient activation. The mice were housed in a pathogen-free facility, and all experiments were performed according to the Canadian Council on Animal Care (CCAC) regulations.

### Acute muscle injury

The tibialis anterior (TA) muscle of 8–12-week-old control or experimental mice was injected with the myotoxin notexin (7 μl), as previously described [37].

### Flow cytometry/FACS

Skeletal muscle tissue was prepared as described previously [37]. Cell preparations were then incubated with primary antibodies for 30 min at 4 °C in supplemented PBS containing 2 mM EDTA and 2 % fetal bovine serum at ~3 × 10 [7] cells per milliliter. We used the following monoclonal-conjugated primary antibodies: anti-platelet endothelial cell adhesion molecule (PECAM)-1 (CD31) (clones MEC13.3, Becton Dickinson, and 390, Cedarlane Laboratories); anti-protein tyrosine phosphatase receptor type C (PTPRC) (CD45) (clone 30-F11, Becton Dickinson); anti-lymphocyte antigen 6A/E (LY6A/E) (Sca-1) (clone D7, eBioscience); anti-vascular cell adhesion molecule (VCAM) (produced in-house); and anti-integrin alpha-7 (produced in-house). Satellite cells were identified as PECAM−, PTPRC−, LY6A/E−, VCAM+, and integrin alpha-7+. Antibody dilution and staining volume were determined experimentally. Where necessary, biotinylated primary antibodies were detected using streptavidin coupled to phycoerythrin (PE), allophycocyanin (APC), phycoerythrin-cyanine 7 tandem complex (PE-Cy7), or fluorescein isothiocyanate (FITC) (Caltag). To assess viability, cells were stained with propidium iodide (1 μg/ml) and Hoechst 33342 (2.5 μg/ml) and resuspended at ~1 × 10 cells/ml [7] immediately before flow cytometry analysis or sorting.

Analysis was performed on LSRII (Becton Dickinson) equipped with three lasers. Data were collected using FACSDiva software. Sorts were performed on a FACS Influx (Becton Dickinson) or FACSAria (Becton Dickinson), both equipped with three lasers, using a 100-μm nozzle at 18 psi to minimize the effects of pressure on the cells. Sorting gates were strictly defined based on “fluorescence minus one” stains.

### Cell culture and immunocytochemistry

Viable single myofibers were isolated from the extensor digitorum longus (EDL) muscle of 6–8-week-old mice following dissociation with collagenase I as previously described [38]. Myofibers and their associated satellite cells were maintained ex vivo for up to 72 h in high-glucose Dulbecco’s modified Eagle’s medium (DMEM) supplemented with 20 % *v*/*v* FBS, 0.5 % *v*/*v* chick embryo extract, and pen-strep. Following culture, single myofibers were fixed in 4 % PFA and then stained overnight with the following primary antibodies: mouse anti-Pax7 (Developmental Studies Hybridoma Bank (DSHB)), mouse anti-MyoD (Dako, clone 5.8A), mouse anti-Myogenin (DSHB, clone FD5).

Fluorescence-activated cell sorting (FACS)-sorted cells were grown in high-glucose DMEM, supplemented with 2.5 ng/ml bFGF (Invitrogen) 20 % *v*/*v* FBS, and 10 % *v*/*v* horse serum. This medium is hereafter referred to as “growth medium.” Cells were seeded in tissue culture-treated plastics coated with Matrigel (BD Biosciences). The media were changed every 24–48 h. To induce myogenic differentiation, confluent myoblasts were cultured in DMEM supplemented with 5 % horse serum for up to 96 h, before being fixed in 4 % PFA and stained overnight with mouse anti-Myosin (DSHB, clone MF20).

### Histology

TA muscles were dissected from mice, fixed in 4 % paraformaldehyde overnight followed by 70 % ethanol overnight, and then embedded in paraffin following standard protocols. Tissues were cut with a microtome in a cross-sectional orientation through the entire length of the muscle. Cross sections of 5 mm thickness were then mounted onto glass slides (Thermo Fisher Scientific, USA) and stained with Masson’s trichrome or Picrosirius red following standard protocols. The cross-sectional area was used as a measure of myofiber size, which was produced by semi-automated measurements on stitched whole-section images (Nikon).

### Gene deletion efficiency measured by allele count

At least 10,000 FACS-purified satellite cells per sample were used for measuring the efficiency of *Ehmt2* conditional knockouts. Cells were lysed and purified for genomic DNA, of which 50 ng per sample was mixed with digital droplet PCR supermix (Bio-Rad) and two TaqMan Copy Number Assays for a duplex (fluorescein amidite (FAM) and VIC) digital droplet PCR assay. The “functional assay” is a TaqMan Copy Number Assay with FAM dye (Thermo Fisher Scientific #4400291 Mm00466045_cn) that detects *Ehmt2* in a region from intron 14 to exon 15, which is found only in the *wildtype* or *floxed* alleles (functional alleles) of the gene. The “reference assay” is a TaqMan Copy Number Assay with VIC dye (Thermo Fisher Scientific #4400291 Mm00466690_cn) that detects *Ehmt2* in a region from intron 25 to intron 26, which is found in any *null*, *wildtype*, or *floxed* alleles of the gene. Droplets of the mixture were generated according to standard digital droplet PCR protocol (Bio-Rad) and ran in a thermocycler for 40 PCR cycles. PCR products were read droplet-wise in duplex (FAM and VIC) following standard protocol (Bio-Rad), and a signal ratio was calculated by dividing the absolute copy number of the functional assay to the copy number of the reference assay. The signal ratio was then used to interpolate the functional allele frequency (%) from a known standard curve (see Additional file 1: Figure S1). The standard curve of signal ratios was produced by performing digital droplet polymerase chain reaction (ddPCR) using genomic DNA that were mixed at known proportions from *Ehmt2*^*wt*/*wt*^ and *Ehmt2*^*null*/*null*^ mouse embryonic fibroblasts. The standard curve has a Pearson coefficient of 0.993, and a statistical test of linearity yielded a *p* value of 0.0001. The results of this measurement are presented either as the frequency of functional alleles in each experimental sample or as the efficiency of gene deletion, which is calculated as the reciprocal of the functional allele frequency.

### Statistics

Mouse weight measurements plotted against age were subjected to linear regression analysis. A sum-of-squares *F* test was performed on a shared model to test the null hypothesis that one curve fits all groups. The resulting *p* value was used to conclude the differences in growth pattern between the groups. Error for mean of means is propagated by weighted pooled variance.

## Results

### Ehmt2 (G9a) is dispensable in skeletal muscle development

**Fig. 1:**
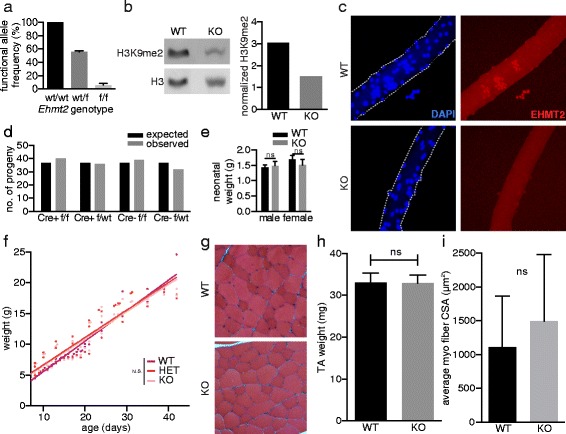
*Ehmt2* is dispensable for normal muscle development. **a** *Ehmt2* deletion efficiency as measured by functional allele frequency in FACS-purified satellite cells. Analysis by gDNA allele counting using ddPCR, *n* ≥ 3. **b** Relative abundance of H3K9me2 in whole skeletal muscle tissue lysate of wildtype and knockout mice, normalized to histone H3. **c** Immunofluorescence detection of EHMT2 on myofiber. **d** Number of live births from *n* ≥ 3 mating pairs of *Myod* ^*Cre*^ *Ehmt2* ^*floxed*/*floxed*^ mice. **e**, **f** Neonatal weight and growth curve of wildtype and knockout mice at D_0_ − D_1_. **g** Masson’s trichrome stain of histological sections of the tibialis anterior muscle of adult mice. **h** Whole muscle weight. **i** Myofiber size measurement by cross-sectional area

To examine the role of *Ehmt2* in myogenesis in vivo, we first established a transgenic mouse model in which the *Ehmt2* gene was conditionally knocked out in the skeletal muscle lineage. Mice harboring loxP-flanked *Ehmt2* alleles (*Ehmt2*^*floxed*^) were crossed to mice with a Cre recombinase gene knocked in to the *Myod* locus (*Myod*^*Cre*^) [34]. To verify the efficiency of the conditional knockout, we performed an exon-specific allele-counting assay using digital droplet PCR to measure functional allele frequency [19] (see the “Methods” section for details). In FACS-purified satellite cells from control mice (*Myod*^*wt/wt*^*Ehmt2*^*floxed*/*floxed*^), the *Ehmt2* functional allele frequency was 100 %; whereas in the knockout mice (*Myod*^*wt*/*Cre*^*Ehmt2*^*floxed*/*floxed*^), the functional allele frequency was reduced to 2.9–7.9 % (95 % CI) (Fig. 1a). These genomic results were consistent with immunostaining quantification of EHMT2 protein in satellite cells. In wildtype mice, EHMT2 was robustly expressed in activated satellite cells whereas no detectable staining was present in the knockout mice (Fig. 1c). Furthermore, western blot analysis of whole skeletal muscle lysates from the conditional knockout mice showed reduction of H3K9me2 levels compared to those of the wildtype (Fig. 1b), congruent with previous reports that H3K9me2 is diminished in *Ehmt2*^*null*/*null*^ models [14].

Knockout and control group progenies from *Myod*^*Cre*^ and *Ehmt2*^*floxed*^ breeding were born at expected Mendelian frequencies (Fig. 1d), and neonatal weights were similar between both groups (Fig. 1e). Growth patterns of knockout and control group progenies were charted by body weight, which showed no statistically significant differences (Fig. 1f). Mature skeletal muscles in these mice are of similar size (Fig. 1g) and showed no difference upon histological examination, which included a comparison of myofiber size by measuring the cross-sectional area of myofibers (Fig. 1h, i). These findings indicate that *Ehmt2* in the skeletal muscle lineage is dispensable in embryonic and fetal development.

### Ehmt2 (G9a) knockout satellite cells have normal proliferation kinetics and differentiation capacity in vitro

**Fig. 2:**
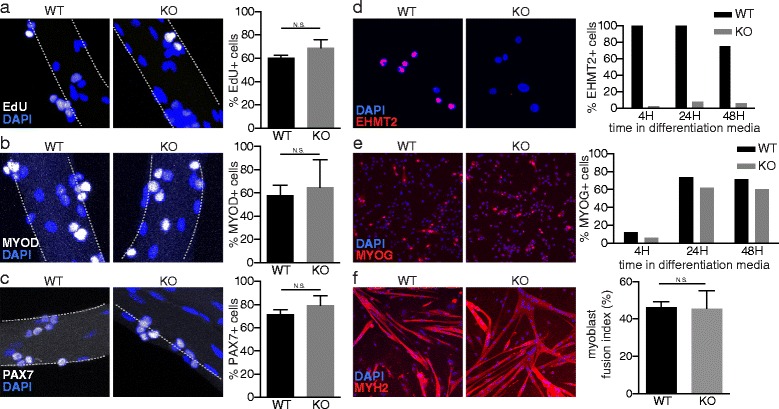
*Ehmt2* knockout satellite cells proliferate and differentiate normally. Myofibers were isolated from WT and Myod-Cre *Ehmt2* KO mice and cultured under growth conditions for 72 h and satellite cells visualized by confocal microscopy. **a** Detection of proliferating cells after 4 h of EdU treatment. **b**, **c** Immunofluorescence detection of MYOD and PAX7. Myoblasts were seeded at equal densities and induced with differentiation media for 4, 24, and 48 h. **d**, **e** Immunofluorescence detection of EHMT2 and MYOG, respectively. **f** Immunofluorescence detection of myosin heavy chain in myotubes, myoblast fusion index calculated as % nuclei inside myosin-expressing myotubes

As previous reports have suggested that *Ehmt2* is an important regulator of C2C12 myogenesis, we next assessed whether it plays a similar role in satellite cell and primary myoblast cultures ex vivo. To analyze satellite cell proliferation, we performed a 4-h 5-ethynyl-2′-deoxyuridine (EdU) pulse on myofiber-associated satellite cells from the wildtype and conditional knockout mice after 72 h in culture. No differences in EdU incorporation were detected between the control and knockout groups (Fig. 2a, e). Similarly, quantification of immunofluorescent staining showed similar numbers of PAX7+ (Fig. 2c) and MYOD+ (Fig. 2b) satellite cells after 72 h in culture. These results indicate that satellite cells lacking *Ehmt2* show comparable rates of proliferation and myogenic activation to wild-type cells ex vivo.

Next, we assessed the requirement of *Ehmt2* for satellite cells to undergo myogenic differentiation. Satellite cell-derived myofibers from wildtype (WT) and knockout (KO) mice were expanded to confluence, induced to differentiate, and then analyzed by immunostaining of MYOG and myosin heavy chain after 4, 24, and 48 h. Under differentiating conditions, MYOG-expressing cells increased, but no significant differences in the percentages of cells expressing MYOG were observed between control and KO myoblasts (Fig. 2e). Similarly, we found no significant differences in myogenic fusion index (ratio of fused nuclei found in myosin-expressing cells to total nuclei) following 48 h of differentiation (Fig. 2f), providing further support that deletion of *Ehmt2* does not have significant effects on the progress or timing of myogenic differentiation in primary myoblasts.

Together, these data provide little evidence that *Ehmt2* plays a major role in the regulation of satellite cell proliferation or myogenic differentiation in vitro.

### Skeletal muscle- and satellite cell-specific deletion of Ehmt2 (G9a) has little effect on muscle regeneration in vivo

Before evaluating the requirement of *Ehmt2* in the response of skeletal muscle to acute injury in vivo, we first analyzed the expression of the gene in our injury model in WT mice, which involved an intramuscular injection of notexin (a snake venom toxin) in the TA muscle. During the ensuing regenerative process, we performed transcriptome sequencing of WT primary myoblasts at different timepoints; in our results, neither *Ehmt2* nor *Ehmt1* showed any dynamic changes in expression during the process, in contrast to key myogenic regulators (Additional file 1: Figure S2).

**Fig. 3:**
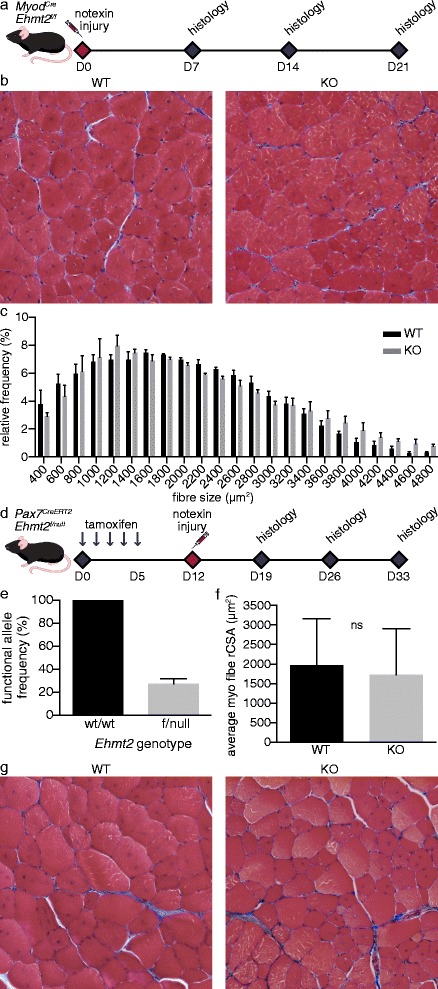
*Ehmt2* is dispensable for muscle regeneration. **a** Schematic diagram of leg injury timeline for *Myod* ^*Cre*^ *Ehmt2* ^*floxed*/*floxed*^ mice. **b**, **c** Masson’s trichrome stain of histological sections of the tibialis anterior muscle of adult *Myod* ^*Cre*^ *Ehmt2* ^*floxed*/*floxed*^ mice at 21 days after injury and myofiber size measurement by cross-sectional area, respectively. **d** Schematic diagram of leg injury timeline for *Pax7* ^*CreERT2*^ *Ehmt2* ^*floxed*/*null*^ mice. **e** *Ehmt2* deletion efficiency by *Pax7* ^*CreERT2*^ as measured by functional allele frequency in FACS-purified satellite cells. Analysis by gDNA allele counting using ddPCR, *n* ≥ 3. **f**, **g** Myofiber size measurement by cross-sectional area and Masson’s trichrome stain of histological sections of the tibialis anterior muscle of induced adult *Pax7* ^*CreERT2*^ *Ehmt2* ^*floxed*/*floxed*^ mice at 21 days after injury, respectively

Then, we evaluated the aforementioned *Myod*^*Cre*^*Ehmt2*^*floxed*/*floxed*^ model, which deletes *Ehmt2* in all skeletal muscles and satellite cells during their development [39]. Following injury, we quantified the cross-sectional area of centrally nucleated myofibers as a measure of regeneration [40]. Despite a trend toward an increased number of the largest fibers in KO samples, no statistically significant difference was found between the control and KO mice in the distribution of myofiber size at 7, 14, or 21 days post injury, indicating comparable regenerative capacities (Fig. 3b, Additional file 1: Figure S3).

*Myod*-driven CRE leads to target deletion early in development and could therefore trigger compensatory effects that mask the regulatory role of *Ehmt2* in adult regenerative myogenesis. To mitigate this risk, we performed a satellite cell-specific, inducible *Ehmt2* KO using a strain carrying the tamoxifen-activated *CreERT2* recombinase knocked in to the *Pax7* locus [35]. This allowed us to confine the gene deletion within adult satellite cells and to use a *Rosa26*^*YFP*^ reporter to monitor the efficiency of induction. One week after the end of tamoxifen treatment, we found that the YFP reporter was activated in 70 % of cells in the satellite cell population (Additional file 1: Figure S4). Since this was not a definitive measure of gene deletion, we further performed *Ehmt2* allele count and found that the functional allele frequency had been reduced to 27.2 ± 4.2 % (SEM) in the satellite cell population purified by FACS.

Acute muscle injury by notexin was performed at 7 days after the final *CreERT2* induction on mice harboring *Pax7*^*CreERT2*^ and *Ehmt2*^*floxed*/*null*^ alleles. At 7, 14, and 21 days after the injury, no significant differences in myofiber size distribution were observed between the control and KO groups (Fig. 3f, Additional file 1: Figure S5, data not shown), suggesting that lack of *Ehmt2* in adult satellite cells does not significantly affect repair and regeneration in vivo.

Together, our data provide little evidence for a role of Ehmt2 in regulating skeletal muscle development, homeostasis, or regeneration in vivo.

## Discussion

In this study, we provide a comprehensive assessment of the biological consequences of G9a deletion in skeletal muscle progenitors. Our data strongly suggests that *Ehmt2* is dispensable for both developmental and regenerative myogenesis in vivo and is not required for normal satellite cell proliferation and myogenic differentiation in vitro.

Although germ line deletion of *Ehmt2* is embryonically lethal, conditional KO models suggest that *Ehmt2* plays distinct roles in different tissues. Studies have shown an involvement of *Emht2* in the regulation of embryogenesis [14, 28, 41], cardiac morphogenesis [16], lymphopoiesis [17, 18], myelopoiesis [19], germ cell development [15], brain and cognitive development [21, 22], and drug addiction [20], confirming *Ehmt2* is capable of controlling a diverse range of biological processes. In the case of myogenesis, we showed that conditional loss of *Ehmt2* in vivo does not induce any significant developmental impact or any significant alterations to the regenerative capacity of myogenic progenitors in response to skeletal muscle injury. Deletion of *Ehmt2* in primary myoblasts also fails to induce any significant alterations in proliferative kinetics or differentiation capacity in vitro, suggesting little role for Ehmt2 in regulating myogenesis.

These results are surprising given the previous reports suggesting an important role for *Ehmt2* in negatively regulating myogenic differentiation of C2C12 cells, an immortalized myogenic line. In particular, it was demonstrated that siRNA knockdown of *Ehmt2* led to enhanced and/or premature differentiation of C2C12 myoblasts [31]. It was further reported that *Ehmt2* is an integral component of the mechanism with which *Bhlhe41*/*Sharp1* regulates in myogenesis, in that it is recruited by SUMOylated BHLHE41/SHARP1 [33], methylates MYOD at lysine 104 [31], and leads to repressive H3K9me2 modifications on *Myod* targets [32]. These results were not consistent with observations in the current study when examining the differentiation capacity of primary myoblasts lacking *Ehmt2*, which showed no premature differentiation, no alterations in myogenic fusion, and normal proliferation. This discrepancy in findings could stem from the fundamental differences between the biological models being analyzed; unlike C2C12 cells, which were derived from a different strain of mouse (C3H) [42], immortalized [43], and have a much shorter doubling time [44], the primary myoblasts in our study were not serially passaged and were analyzed in their myofiber niche during proliferation and on Matrigel during differentiation, in addition to in vivo analyses. These differences may be particularly relevant in the case of *Ehmt2*, as we have recently reported, in another tissue system, that its absence has drastically different effects on transformed compared to natural hematopoietic cells [19].

To date, no genome-wide analysis of the *Ehmt2*-mediated H3K9me2 in myogenic cells exists. Dynamic changes in H3K9me2 have been reported at specific gene bodies and regulatory regions [45, 46], suggesting that modulating this epigenetic mark may affect gene transcription. However, in our experiments, lack of *Ehmt2* led to a dramatic drop in the global levels of detectable H3K9me2 in the absence of any effects on skeletal muscle development. This indicates that *Ehmt2* activity is mostly non-redundant and questions the importance of *Ehmt2*-mediated histone modifications in myogenesis in particular and in the control of differentiation in general.

What remains unclear is the status of EHMT2-mediated methylation of MYOD at lysine 104 [31] in the in vivo model. Ling et al. reported the identification of this residue by mass spectrometry of peptides resulting from the digestion of MYOD with trypsin [31]. However, the reported MYOD peptides, ACKACKRKTT and its methylated forms, do not appear to be obtainable by trypsin digestion alone or by any commonly used digestion method. The reported MYOD peptide is also not found in the tandem mass spectrometry proteomics repository PeptideAtlas (https://db.systemsbiology.net/sbeams/cgi/shortURL?key=1mk36ybs). More intriguingly, the proposed mechanism [31] was based on liquid chromatography–mass spectrometry (LC-MS) results, showing that the different methylation states of the MYOD peptide are separated by only 1 m/z unit each. Methylation adds 14 Da to the peptide mass; thus, each peptide would have to carry 14 charges on 10 residues. The LC-MS results could not possibly correspond to methylation of the reported MYOD peptide. The uncertainty of MYOD methylation, together with the dispensability of Ehmt2-mediated H3K9me2 in vivo, casts doubts on the proposed role of Ehmt2 in *Bhlhe41*/*Sharp1*-mediated regulation of myogenesis [32]. Nevertheless, SUMOylated *Bhlhe41*/*Sharp1* [33] may still regulate *Myod* and downstream targets through an alternative mechanism.

Although we have shown here that the loss of EHMT2’s histone methylation function in our model was not compensated, the possibility exists that its potential interaction with myogenic regulators could be compensated by another gene, such as its close homologue *Ehmt1* (*GLP*). These two genes are highly similar in structure, as their protein products contain highly similar catalytic domains for lysine methylation (SET domain) and a set of ankyrin repeats for protein–protein interaction. These two enzymes are known to form heterodimers [14] but also play unique roles depending on the cell type and developmental stage [47]. Using domain-specific mutations, the EHMT1 but not the EHMT2 ankyrin repeats were found to be required for mouse viability [48], suggesting that this domain function in EHMT2 could be compensated by EHMT1. On the other hand, the SET domain in EHMT1 is dispensable for mouse viability [16], suggesting EHMT2 could compensate for EHMT1’s methyltransferase activity. In our *Ehmt2* KO cells, even though the histone methyltransferase activity, a function shared by both proteins, is not compensated by EHMT1, its ankyrin repeat-dependent protein–protein interactions may be compensated. Both proteins have been reported to methylate a number of non-histone targets beyond MYOD [49, 50], and it is unknown if EHMT1 could replace EHMT2 in binding and methylating these targets. Interestingly, such compensation by *Ehmt1* was not observed in C2C12 studies [31], and in our transcriptome data, neither of the genes showed any dynamic changes during regeneration. Nevertheless, to fully address this concern, a conditional double KO of *Ehmt2* and *Ehmt1* in myogenesis would be required.

## Conclusions

In this study, we analyzed tissue-specific KO models of *Ehmt2* both during development and in adult satellite cells, and unlike as previously reported, we found no evidence for a significant role of this methyltransferase in the development or regeneration of skeletal muscle. The drop in H3K9me2 levels observed in cells lacking Ehmt2 strongly suggests that this histone modification is dispensable for the regulation of myogenesis. Primary cultures revealed that Ehmt2 does not significantly alter the proliferation and differentiation processes of satellite cells. Thus, the proposed regulatory role of Ehmt2 in myogenesis cannot be validated in vivo.

## Additional file

Additional file 1: Main figures are referred to as Fig. # in the article. Figure legends are supplied on the next page. Supplementary figures with figure legends are referred to as Supplementary Fig. # in the article.

## Abbreviations

APC: allophycocyanin
bHLH: basic helix-loop-helix
Bhlhe41: basic helix-loop-helix family member e41, aka Sharp1
CCAC: Canadian Council on Animal Care
ChIP: chromatin immunoprecipitation
CI: confidence interval
Cre: Causes Recombination
ddPCR: digital droplet polymerase chain reaction
DMEM: Dulbecco’s modified Eagle’s medium
DNMT: DNA methyltransferase
DSHB: Developmental Studies Hybridoma Bank
EDL: extensor digitorum longus
EdU: 5-ethynyl-2′-deoxyuridine
Ehmt1: euchromatic histone-lysine *N*-methyltransferase 1, aka GLP (G9a-like protein)
Ehmt2: euchromatic histone-lysine *N*-methyltransferase 2, aka G9a
FACS: fluorescence-activated cell sorting
FAM: fluorescein amidite
FITC: fluorescein isothiocyanate
H2B: histone 2B
H3K27ac: acetylation of lysine 27 on histone H3 protein subunit
H3K4me1: unimethylation of lysine 4 on histone H3 protein subunit
H3K9me2: trimethylation of lysine 9 on histone H3 protein subunit
HP1: heterochromatin protein 1
KO: knockout
LC-MS: liquid chromatography–mass spectrometry
LY6A/E: lymphocyte antigen 6A/E, aka SCA-1 (stem cell antigen 1)
MRF: myogenic regulatory factor
Mrf4: myogenic regulatory factor 4, aka myogenic factor 6 (herculin)
Myf5: myogenic factor 5
Myog: myogenin
Pax7: paired box 7
PE: phycoerythrin
PECAM: platelet endothelial cell adhesion molecule, aka CD31 (cluster of differentiation 31)
PE-Cy7: phycoerythrin-cyanine 7 tandem complex
PTPRC: protein tyrosine phosphatase receptor type C, aka CD45 (cluster of differentiation 45)
SEM: standard error of mean
SET: Su (var)3-9 and enhancer of zeste
Shh: sonic hedgehog
SUMO: small ubiquitin-like modifier
TA: tibialis anterior
VCAM: vascular cell adhesion molecule, aka CD106 (cluster of differentiation 106)
WT: wildtype
