# Supplementary material for: The lysine methyltransferase Ehmt2/G9a is dispensable for skeletal muscle development and regeneration
Source: Skelet Muscle. 2016 May 27;6:22. doi: 10.1186/s13395-016-0093-7 (PMC4882833; doi:10.1186/s13395-016-0093-7)
Supplement: Additional file 1: — Main figures are referred to as Fig. # in the article. Figure legends are supplied on the next page. Supplementary figures with figure legends are referred to as Supplementary Fig. # in the article. [file 13395_2016_93_MOESM1_ESM.pdf]

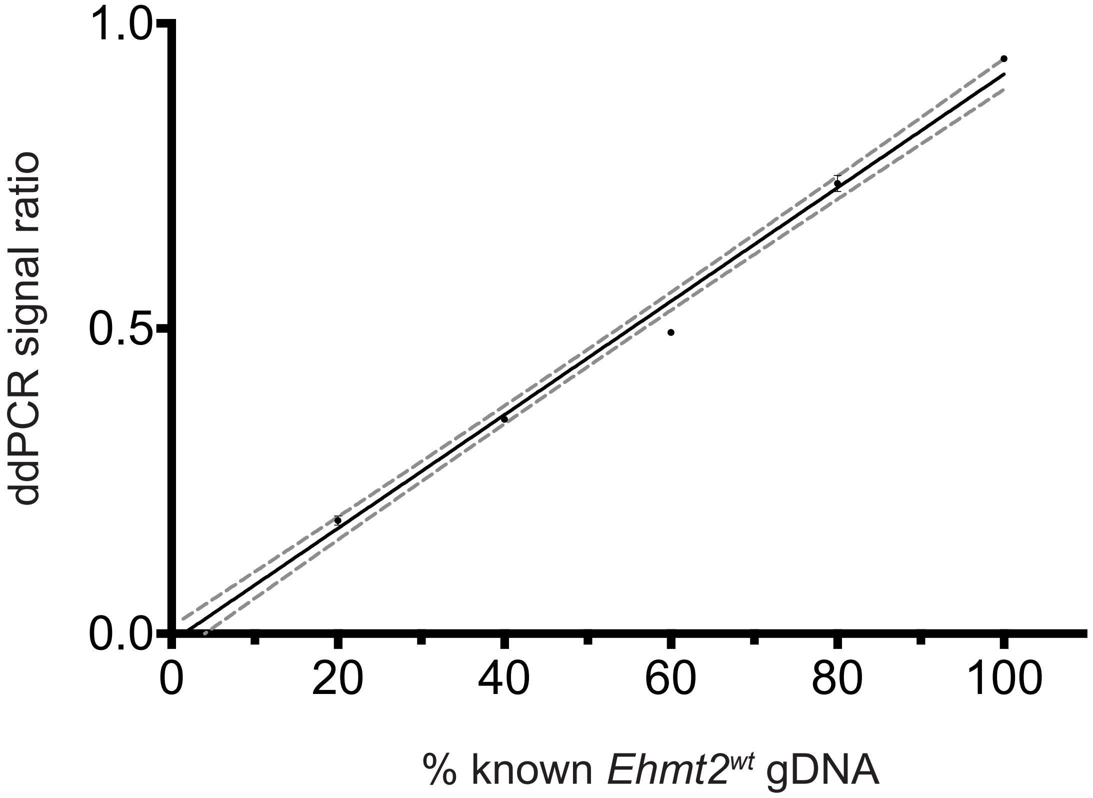

Supplementary Fig. 1. Standard curve of ddPCR signal ratio. Genomic DNA was isolated from known *Ehmt2*$^{wt/wt}$ and *Ehmt2*$^{null/null}$ mouse embryonic fibroblasts, and mixed at known ratios to produce standard samples. The functional allele frequencies of experimental samples were interpolated from this curve. Pearson's r=0.993. Test of linearity p=0.0001.

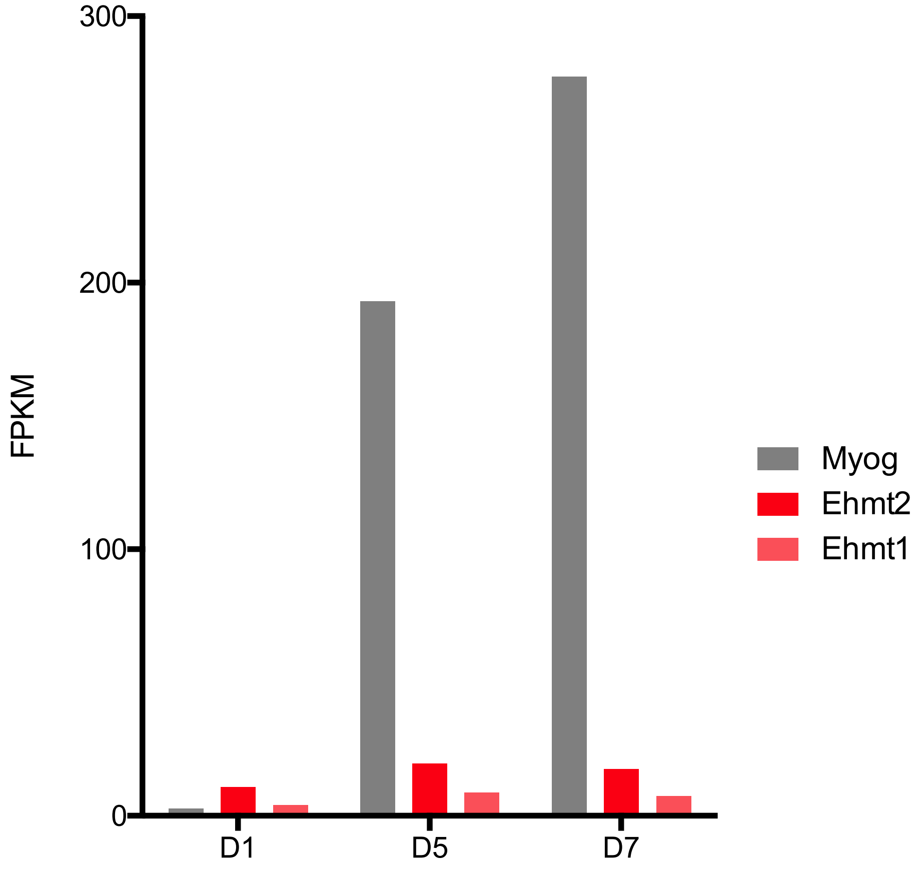

Supplementary Fig. 2. Ehmt2 gene expression in satellite cells during adult skeletal muscle regeneration. Satellite cells were purified by FACS at D1, D5, and D7, after notexin-induced TA muscle injury. Gene expression was analyzed by RNA sequencing (Illumina MiSeq).

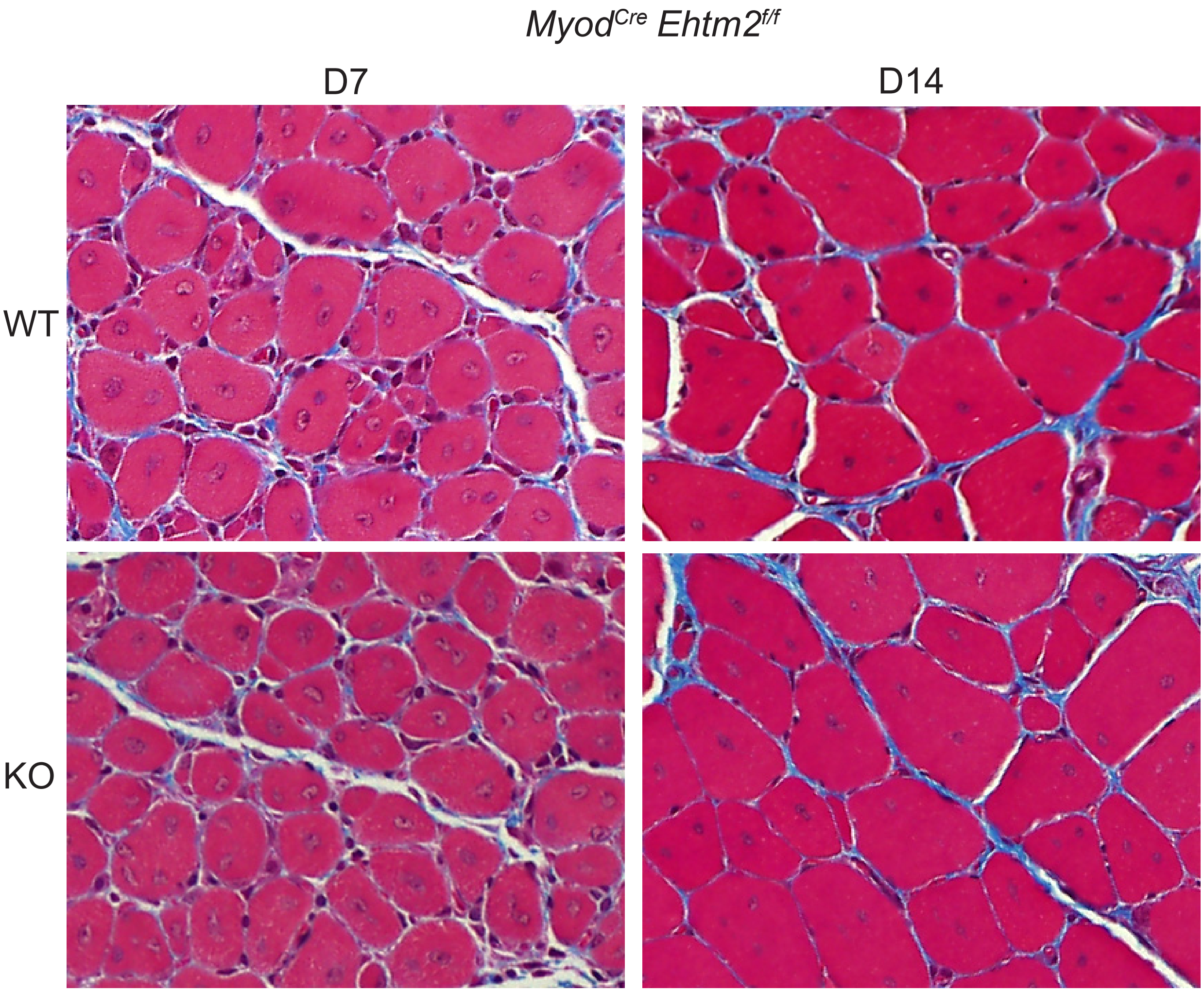

Supplementary Fig. 3. In vivo skeletal muscle regeneration of *Myod*^{Cre} *Ehtm2*$^{f/f}$ knockout mice. Masson’s trichrome stain of histological sections of the tibialis anterior muscle of adult *Myod*^{Cre} *Ehtm2*$^{f/f}$ mice at 7 and 14 days after injury.

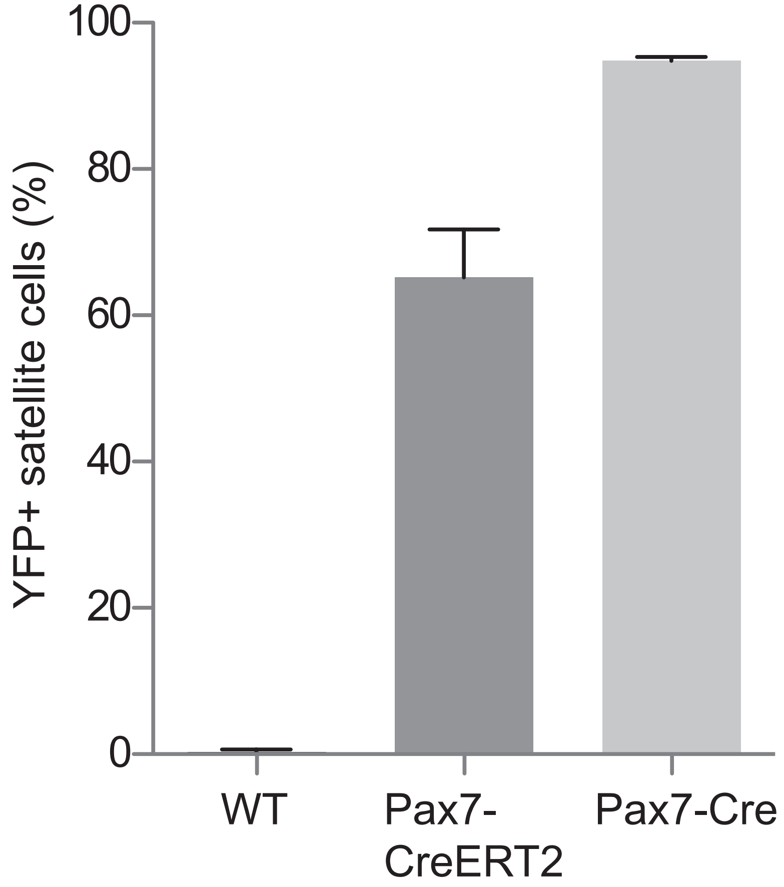

Supplementary Fig. 4. YFP reporter expression as a measure of CreERT2 induction efficiency. A mouse line carrying a tamoxifen-activated CreERT2 recombinase knocked-in to the Pax7 locus (middle), was used for generating inducible conditional knockout. YFP expressing cells were quantified within the satellite cell population by flow cytometry, using surface markers, one week after the end of tamoxifen treatment. Wildtype mice (left) and mice carrying a constitutively active $Pax7^{Cre}$ (right) were used as controls and for comparison.

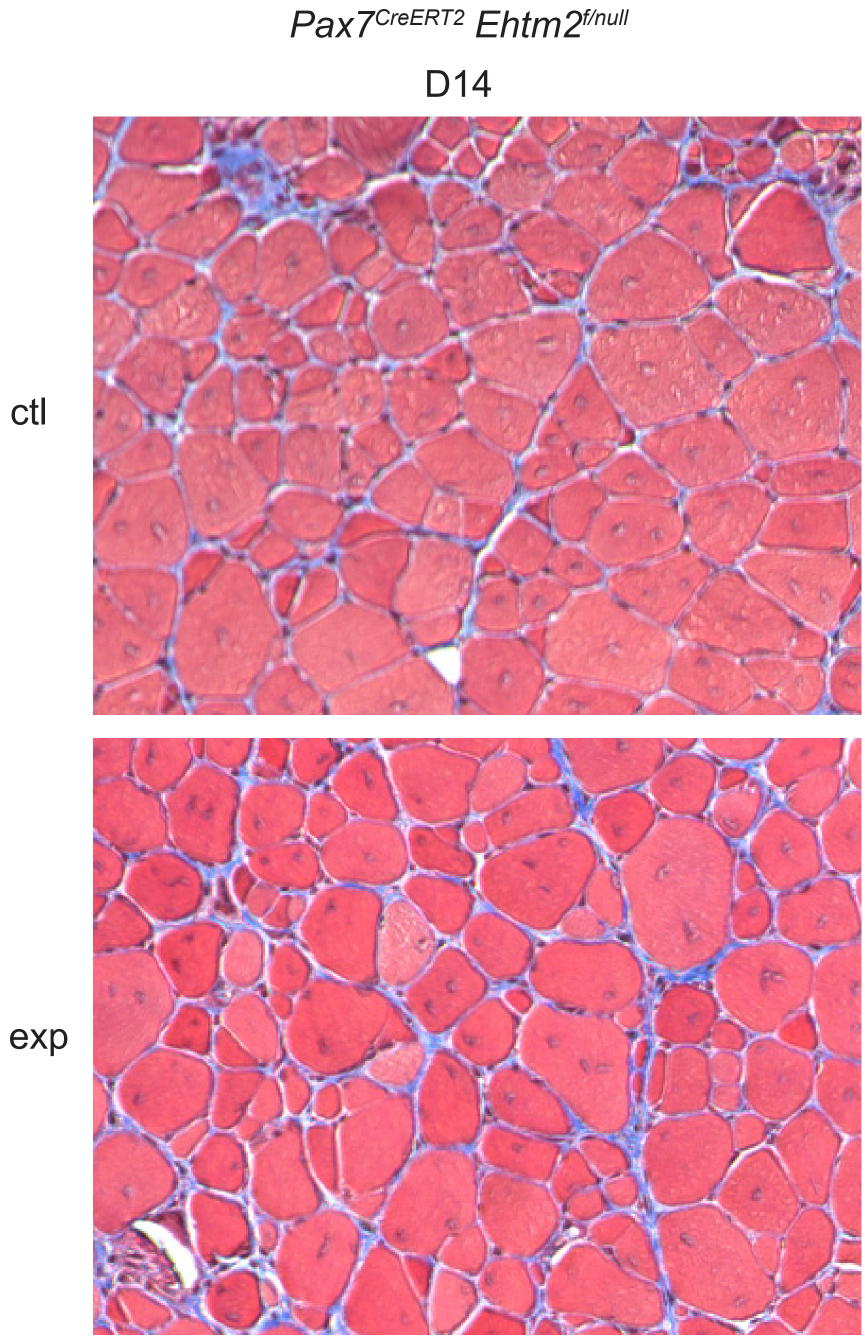

Supplementary Fig. 5. In vivo skeletal muscle regeneration of *Pax7*^{CreERT2} *Ehtm2*$^{f/null}$ knockout mice. Masson's trichrome stain of histological sections of the tibialis anterior muscle of adult *Pax7*^{CreERT2} *Ehtm2*$^{f/null}$ mice at 14 days after injury.
